# POC1A, prognostic biomarker of immunosuppressive microenvironment in cancer

**DOI:** 10.18632/aging.204141

**Published:** 2022-06-23

**Authors:** Qi Zhao, Shuping Gao, Xin Chen, Xiyan Zhu

**Affiliations:** 1 Department of Thoracic Surgery, The Fourth Hospital of Hebei Medical University, Shijiazhuang 050011, China; 2 Department of Pharmacy, The Fourth Hospital of Hebei Medical University, Shijiazhuang 050011, China

**Keywords:** TCGA, POC1A, prognostic biomarker, pan-cancer, tumor-infiltration

## Abstract

POC1 centriolar protein A (POC1A) effect in pan-cancer remains uncertain. The POC1A expression in normal and tumor tissues underwent analysis in this study utilizing data from the Genotype-Tissue Expression (GTEx) project and the Cancer Genome Atlas (TCGA) database. POC1A prognostic value and clinicopathological features were assessed utilizing the TCGA cohort. The relationship between immunological cell infiltration and POC1A of TCGA samples downloaded from TIMER2 and ImmuCellAI databases were observed. The relation between POC1A and immunological checkpoints genes, microsatellite instability (MSI) as well as tumor mutation burden (TMB) was also evaluated. Additionally, gene set enrichment analysis (GSEA) was utilized for exploring POC1A potential molecular mechanism in pan-cancer. In almost all 33 tumors, POCA1 showed a high expression. In most cases, high POC1A expression was linked significantly with a poor prognosis. Additionally, Tumor immune infiltration and tumors microenvironment were correlated with the expression of POC1A. In addition, TMB and MSI, as well as immune checkpoint genes in pan-cancer, were related to POC1A expression. In GSEA analysis, POC1A is implicated in cell cycle and immune-related pathways. These results might elucidate the crucial roles of POC1A in pan-cancer as a prognostic biomarker and immunotherapy target.

## INTRODUCTION

Worldwide, cancer is one of leading mortality causes [1]. Most cancers are diagnosed at a progressive stage, so the cure rate is quite low. Tumor immunotherapy has revolutionized the therapeutic effect of cancer, but the treatment is only beneficial to a small number of cancer patients [2]. According to current studies, tumor microenvironment has a key role in tumor occurrence and progression [3–5]. Tumor microenvironment is fundamentally consisting of cancerous cells, immune cells, various signal molecules, fibroblasts and extracellular matrix, where immune cells are a critical part [6]. Tumor cells secrete immunosuppressive cytokines and reprogram immune cells in the tumor microenvironment; as a result, tumor immune microenvironment is inhibited, so as to escape immune recognition and finally escape immune surveillance [7]. Tumor immunosuppressive microenvironment will not only promote tumor progression, but also weaken the effect of immunotherapy [8]. Hence, it is critical to find innovative biomarkers for identifying tumor immunosuppressive microenvironment to improve the effectiveness of tumor immunotherapy.

As an important component of the centrosome, in biological processes, POC1A (POC1 centriolar protein homolog A, also known as WDR51A, plays a critical role for centrioles formation and steady-state [9]. Numerous studies confirmed the link between POC1A and facial dysmorphism and hypotrichosis (SOFT) syndrome, onychodysplasia, short stature, all of which are associated with abnormal cell mitosis [10, 11]. POC1A may have a crucial role in cell proliferation, based on these studies. Therefore, POC1A is considered to be a cell cycle-regulating factor [12]. At the moment, some studies have explored the POC1A’s role in tumors. Wada et al. revealed that POC1A was a biomarker for predicting the recurrence of intrahepatic cholangiocarcinoma [13]. Dastsooz et al. suggested that POC1A gene might be the new target for cancers therapies [14]. Even so, POC1A’s role in pan-cancer is still uncertain.

Using TCGA database, this is the initial study to accomplish pan-cancer analysis of POC1A. The relationship of POC1A expression with prognosis, tumour immunity microenvironment, DNA methylation, immune checkpoint gene, microsatellite instability (MSI), drug sensitivity and tumour mutation burden (TMB) was systematically observed to elucidate POC1A clinical role and potential molecular mechanism in pan-cancer.

## MATERIALS AND METHODS

### Analysis of gene expression

The tumor immune estimation resource version 2 (TIMER2) database (http://timer.cistrome.org) was utilized for exploring the variations between POC1A expression across different tumor tissues or tissue subtypes as well as adjacent normal tissues acquired from the TCGA project [15]. In the form of a box plot, gene expression levels distributions were represented. The Wilcoxon test was utilized for assessment of statistical significance of differential expression. For tumors without normal control, such as CESC, DLBC, GBM, OV, PAAD, PCPG, SARC, UCS, THYM, LGG, etc., Genotype-Tissue Expression (GTEx) and TCGA databases were utilized to acquire POC1A expression profile data of tumor tissues and matched normal tissues. The R language was employed for analyses and graphics. Subsequently, the tumor stage information of the TCGA database has been utilized for exploring the POC1A expression in various tumor stages. UCSC Xena was utilized to download the TCGA and GTEx expression profiles and clinical information (https://xenabrowser.net/datapages/).

### Gene alteration analysis

The cBioPortal database was utilized for downloading mutation and copy number variation (CNV) data of POC1A (https://www.cbioportal.org/) [16].

### Survival prognosis analysis

For exploring the POC1A expression effect on pan-cancer prognosis, we utilized Kaplan-Meier and Univariate Cox regression (UniCox). The optimal cutoff value was utilized to differentiate the groups of POC1A with low and high expression. Survival analyses (overall, disease-specific, progression-free, and disease-free) were assessed. The R packages “survminer” and “survival” were utilized to analyse the data.

### Immune infiltration analysis

The immunosuppressive microenvironment is one of the reasons contributing to tumor patients’ poor prognosis; therefore, the correlation between POC1A and the immune microenvironment was further explored. The TIMER2 and ImmuCellAI databases were utilized for downloading data of immune cell infiltration of TCGA (http://timer.comp-genomics.org/) (http://bioinfo.life.hust.edu.cn/ImmuCellAI#!/) [17]. The correlation between POC1A and immune cell infiltration was calculated. The R language “estimate” package was utilized to calculate StromalScore, ImmuneScore, and ESTIMATEScore (Sum of StromalScore and ImmuneScore). The correlation between POC1A expression and these scores was evaluated.

### Immune checkpoints genes analysis

Tumor immune regulation is tightly linked to immune checkpoint-related genes. The association between immune checkpoint gene expression and POC1A expression underwent analysis. Additionally, the correlation of POC1A with immune regulatory genes was explored.

### TMB and MSI analysis

Tumor mutation burden (TMB) is linked to immunotherapy effectiveness in various cancers. TMB was computed for each tumor sample, and the relation between TMB and POC1A expression was assessed utilizing Spearman’s correlation. The relationship between MSI and POC1A expression was also analyzed.

### Gene set enrichment analysis (GSEA) of POC1A in pan-cancer

For POC1A expression profile assessment in pan-cancer, the GSEA was utilized relying on the Reactome database. The analysis was implemented in the R package “clusterprofiler”. The top 20 results of each tumor identified by GSEA analysis were displayed.

### POC1A correlation with drug sensitivity analysis in pan-cancer

The Genomics of Drug Sensitivity in Cancer database was utilized for downloading 192 medications IC50 values as well as 809 cell lines’ gene expression profiles (GDSC: https://www.cancerrxgene.org/). The analysis of POC1A correlation with 192 medications IC50 values was done.

## RESULTS

### POC1A is highly expressed in pan-cancer

**Figure 1:**
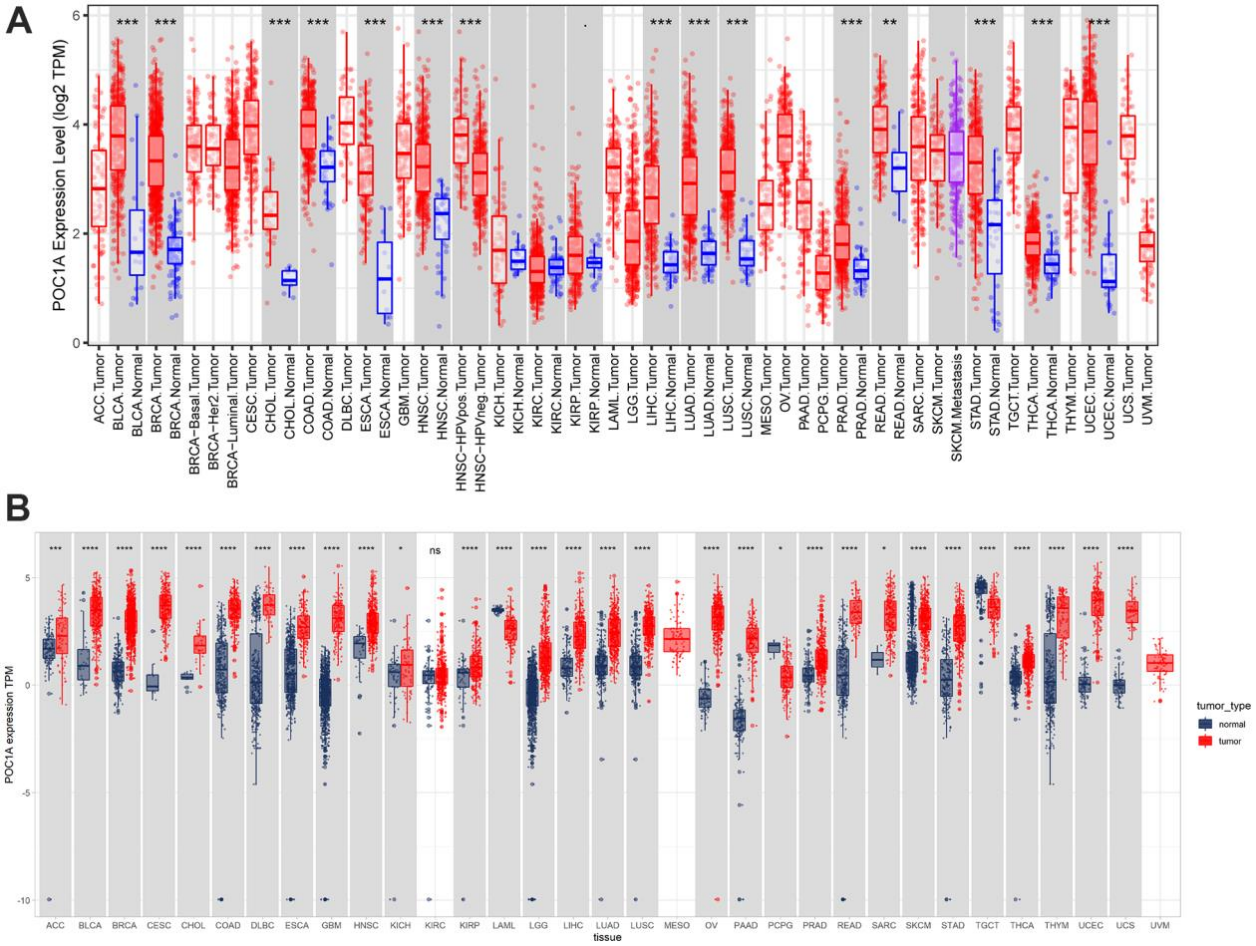
**Pan-cancer POC1A expression.** (**A**) POC1A expression analysis in pan-cancer through TIMER2 database utilization. (**B**) Expression of POC1A in normal and tumor tissues from the GTEx and TCGA cohorts. *P<0.05, **P<0.01, ***P<0.001, ****P<0.0001.

**Figure 2:**
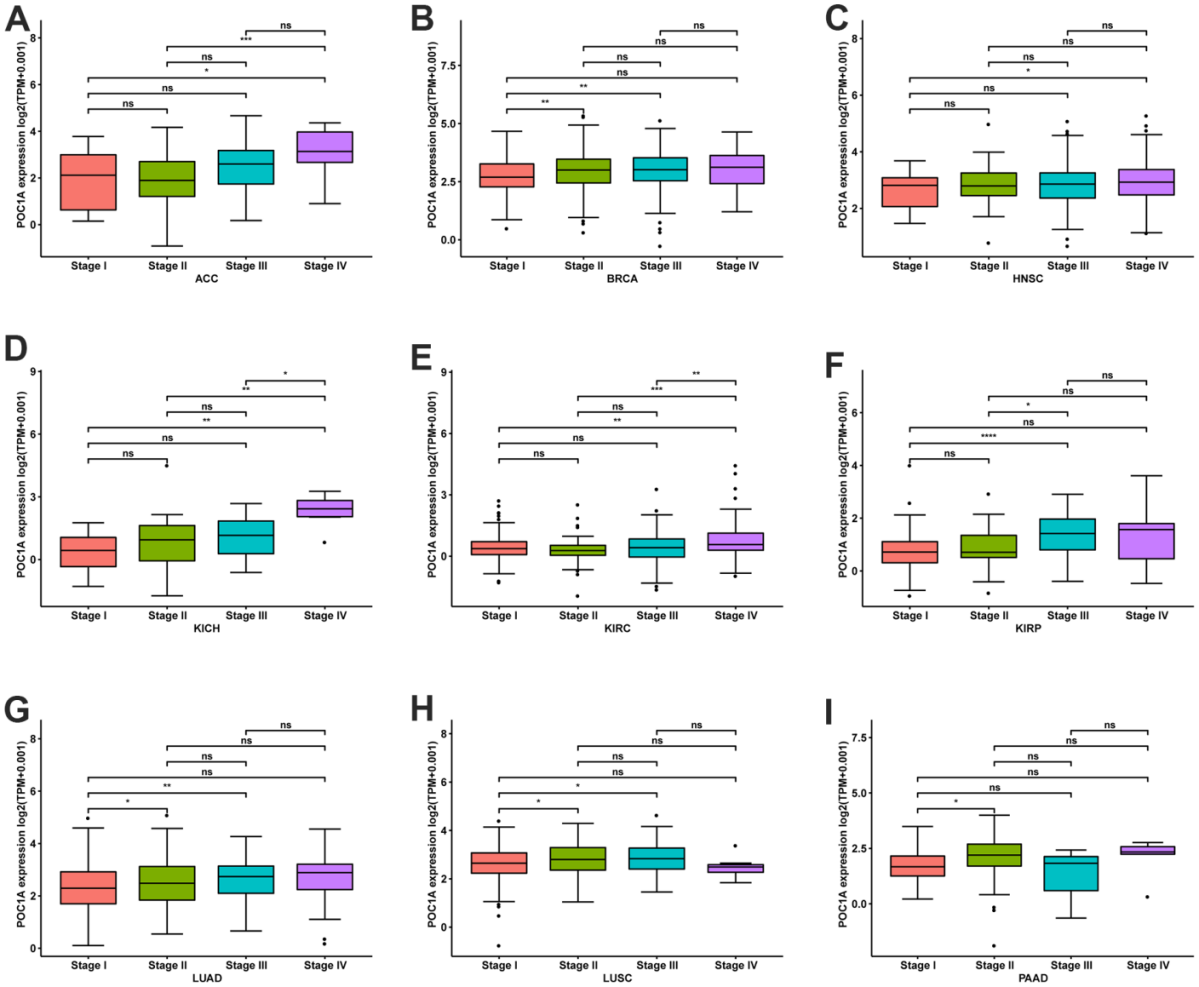
**POC1A expression at various stages of tumor.** (**A**–**I**) Expression of POC1A at various stages of tumor in indicated tumors. *P<0.05, **P<0.01, ***P<0.001, **** <0.0001.

POC1A expression in pan-cancer was observed through TIMER2 webserver usage. As listed in Figure 1A, POC1A expression levels were significantly elevated in tumor tissues of BLCA, BRCA, HNSC, HNSC-HPV, LUAD, CHOL, LUSC, PRAD, STAD, ESCA, THCA, COAD, LIHC, UCEC (P<0.001), READ (P<0.01), and KIRP (P<0.05) than adjacent normal tissues. POC1A expression was assessed using TCGA and GTEx data for tumors without normal control. POC1A overexpression was detected in 27 of 33 types of cancer, comprising ACC, BLCA, BRCA, CESC, CHOL, COAD, DLBC, ESCA, GBM, HNSC, KICH, KIRP, LGG, LIHC, LUAD, LUSC, OV, PAAD, PRAD, READ, SARC, SKCM, STAD, THCA, THYM, UCEC, and UCS. Even so, POC1A under-expression was detected in three tumors, comprising LAML, PCPG, and TGCT (Figure 1B). The correlation of POC1A expression with pathological tumor staging in the TCGA cohort was done, and it was raised as tumor stages increased in ACC, BRCA, KICH, KIRC, LUAD, LUSC, HNSC, PAAD and KIRP (Figure 2A–2I).

### POC1A gene alteration in pan-cancer

**Figure 3:**
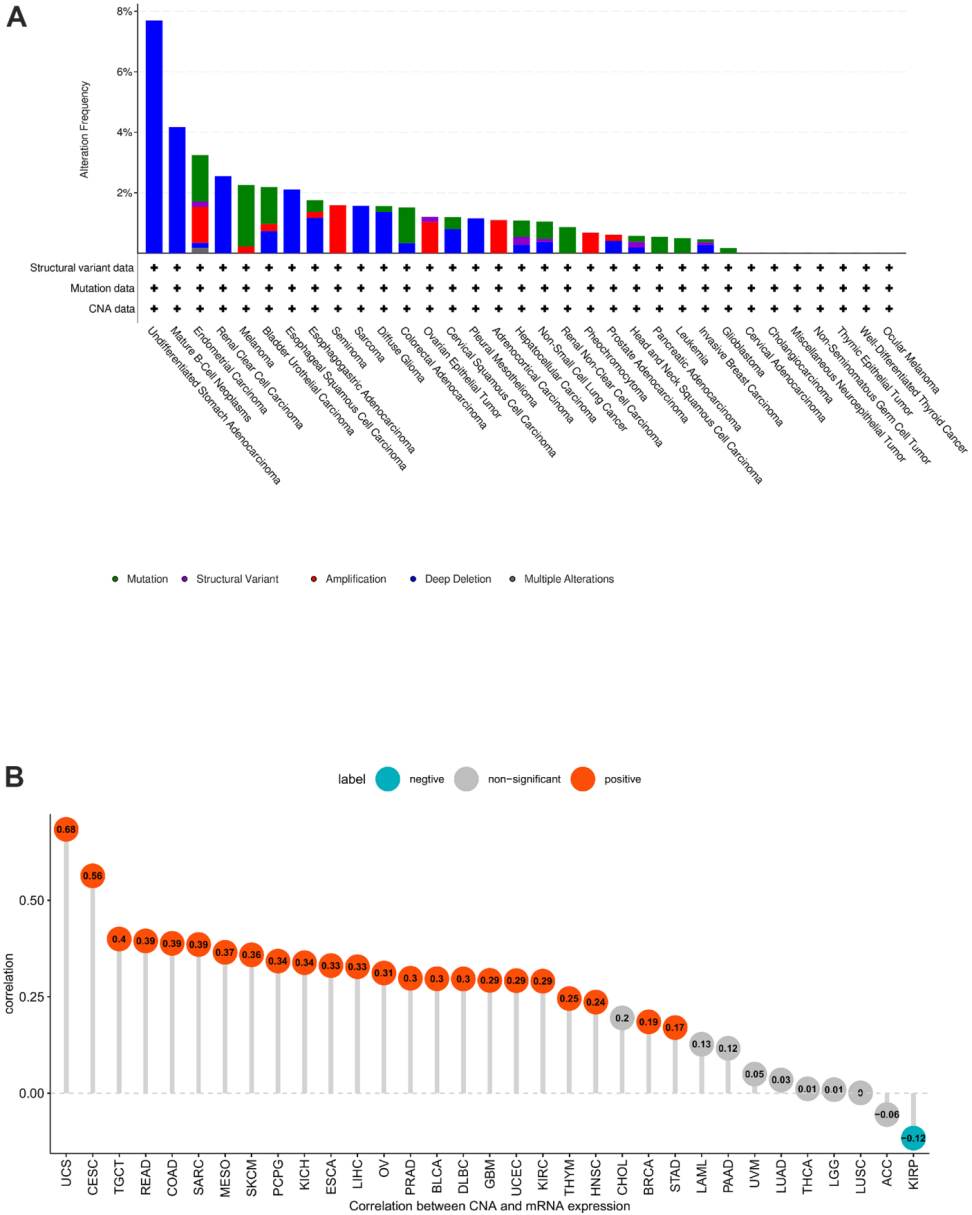
**POC1A gene alteration.** (**A**) POC1A mutation and CNA status in TCGA pan-cancer. (**B**) Correlation of POC1A expression with CNA.

Copy number alteration (CNA) and mutation influence gene expression. Hence, we evaluated the POC1A mutations and CNA. We observed the highest frequency of POC1A alterations (>7%) in patients with undifferentiated stomach adenocarcinoma, where “Mutation” was the major type (Figure 3A). POC1A expression was negatively correlated with KIRP but positively correlated with can in 23 of 33 tumors (Figure 3B), suggesting that high CNA was among the major reasons for high POC1A expression in pan-cancer.

### POC1A high expression in pan-cancer is related to poor prognosis

**Figure 4:**
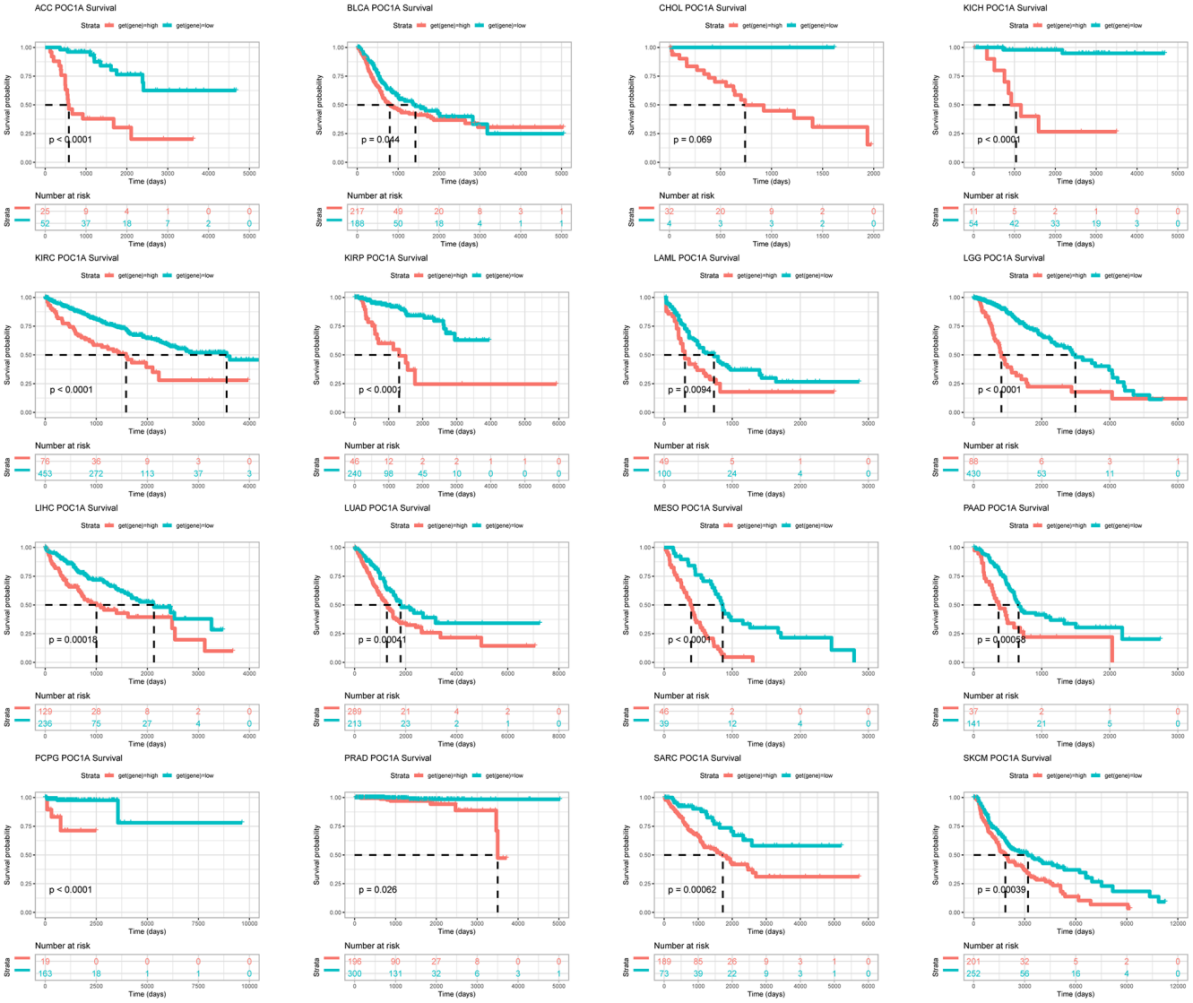
**POC1A prognostic value.** POC1A overall survival analysis via Kaplan-Meier in TCGA pan-cancer in indicated tumors.

**Figure 5:**
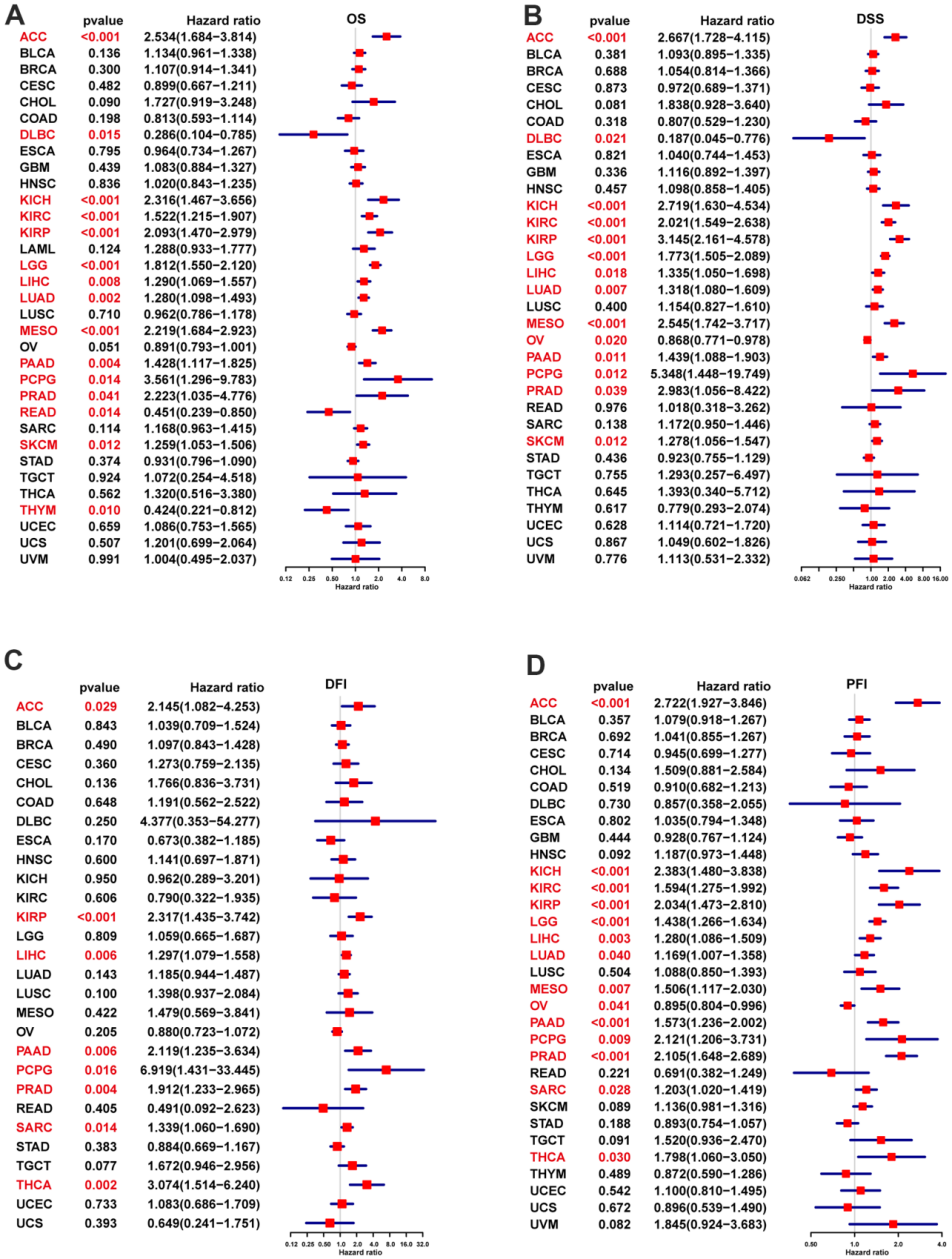
**POC1A UniCox analysis.** (**A**) POC1A overall survival (OS) analysis utilizing the UniCox in TCGA pan-cancer. (**B**) POC1A disease-specific survival (DSS) analysis in TCGA pan-cancer utilizing the UniCox. (**C**) POC1A disease-free interval (DFI) analysis in TCGA pan-cancer utilizing UniCox. (**D**) POC1A progression-free interval (PFI) analysis in TCGA pan-cancer utilizing UniCox. Red color indicates statistical significance.

The UniCox and Kaplan-Meier survival analyses were utilized for exploring POC1A prognostic value in pan-cancer. POC1A low and high expressions were differentiated using the optimal cut-off value. According to Kaplan-Meier, worse overall survival was related to elevated POC1A expression in ACC, BLCA, CHOL, KICH, KIRC, KIRP, LAML, LGG, LIHC, LUAD, MESO, PAAD, PCPG, PRAD, SARC, and SKCM (Figure 4). POC1A was considered to be a risk factor for OS, according to UniCox analysis in ACC, DLBC, KICH, KIRC, KIRP, LGG, LIHC, LUAD, MESO, PAAD, PCPG, PRAD, READ, SKCM, and THYM (Figure 5A). POC1A prognostic value in pan-cancer for DSS, DFI, and PFI was also analyzed, and the results are illustrated in Figure 5B–5D. Based on these findings, elevated POC1A expression in pan-cancer was linked to a poor prognosis and might be a potential prognostic biomarker.

### POC1A correlation in pan-cancer with microenvironment and tumor immune infiltration

**Figure 6:**
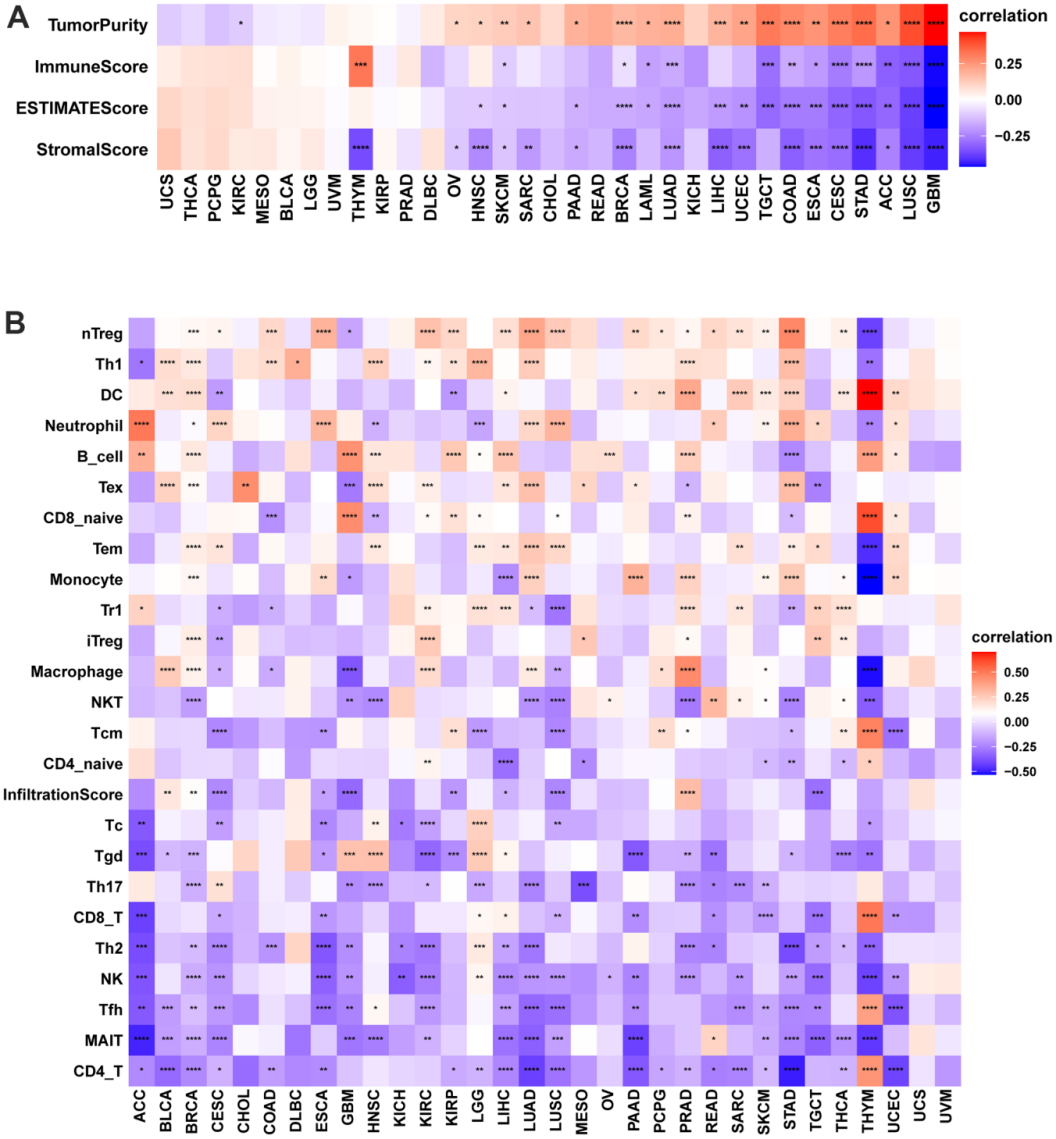
**Immune infiltration analysis according to the ImmuCellAI database.** (**A**) Correlation of POC1A expression with immune cell infiltration in LUAD. (**B**) Relation of POC1A expression with tumor purity, ImmuneScore, ESTIMATEscore and StromalScore. Red and blue colors indicate positive and negative correlations, respectively; deeper color indicates a strong correlation *P<0.05, **P<0.01, ***P<0.001, ****P<0.0001.

**Figure 7:**
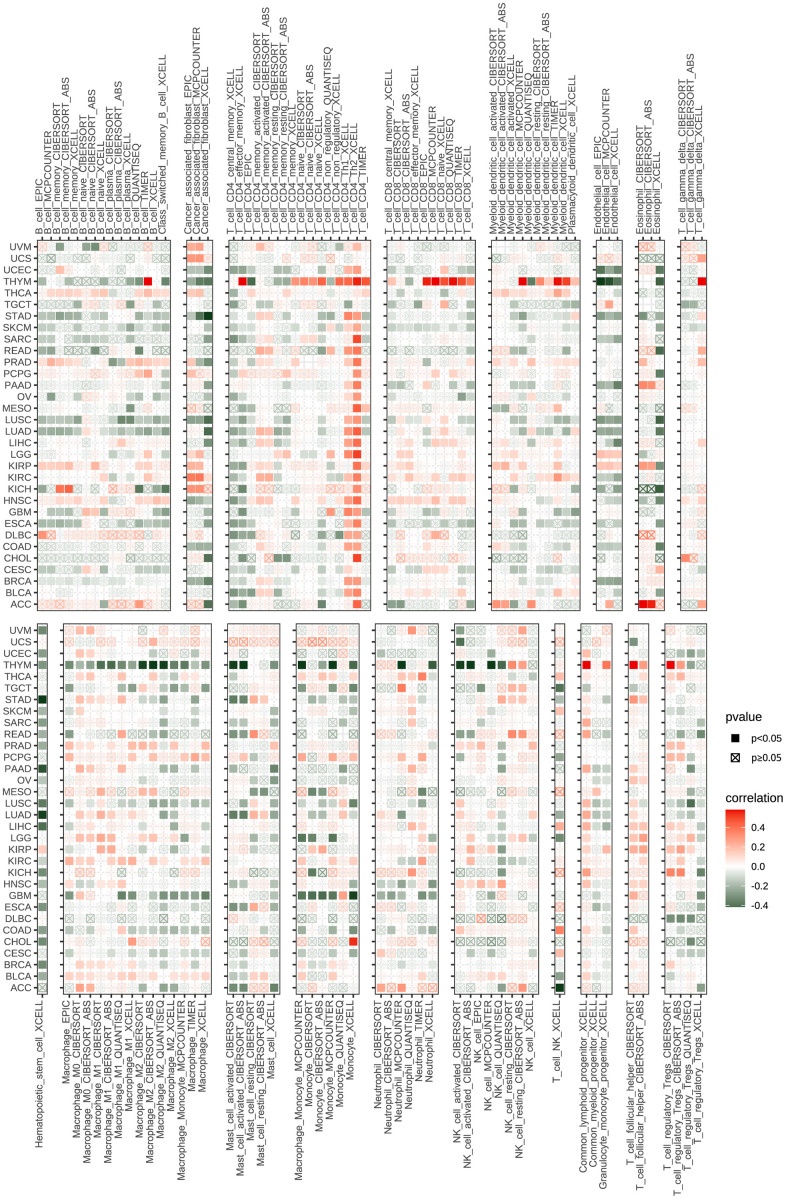
**Immune infiltration analysis according to the TIMER2 database.** In pan-cancer, immune cell infiltration levels are associated with POC1A expression. Red and green colors indicate positive and negative correlation, respectively; deeper color indicates a strong correlation *P<0.05, **P<0.01, ***P<0.001, ****P<0.0001.

The tumor-infiltrating lymphocytes amount is an essential predictor of prognosis in cancer patients and their responsiveness to immunotherapy. The StromalScore, ImmuneScore, and ESTIMATEScore of the tumor tissue were calculated using the R language “estimate” package, and their correlation with POC1A expression was evaluated. The findings revealed that POC1A was shown to be negatively correlated with StromalScore and ImmuneScore in most tumors and positively correlated with tumor purity. (Figure 6A). By exploring the correlation of POC1A expression with immune cell infiltration utilizing ImmuCellAI database, it was noticed that POC1A was positively associated with nTreg cells in most tumors while negatively correlated with immune killer cells as CD4 and CD8 T cells and activated natural killer (NK) cells (Figure 6B). Similarly, according to TIMER2 database results, a negative correlation of POC1A with NK and CD8 T cells was noted in most tumors (Figure 7).

### POC1A expression is associated with immune checkpoint genes

**Figure 8:**
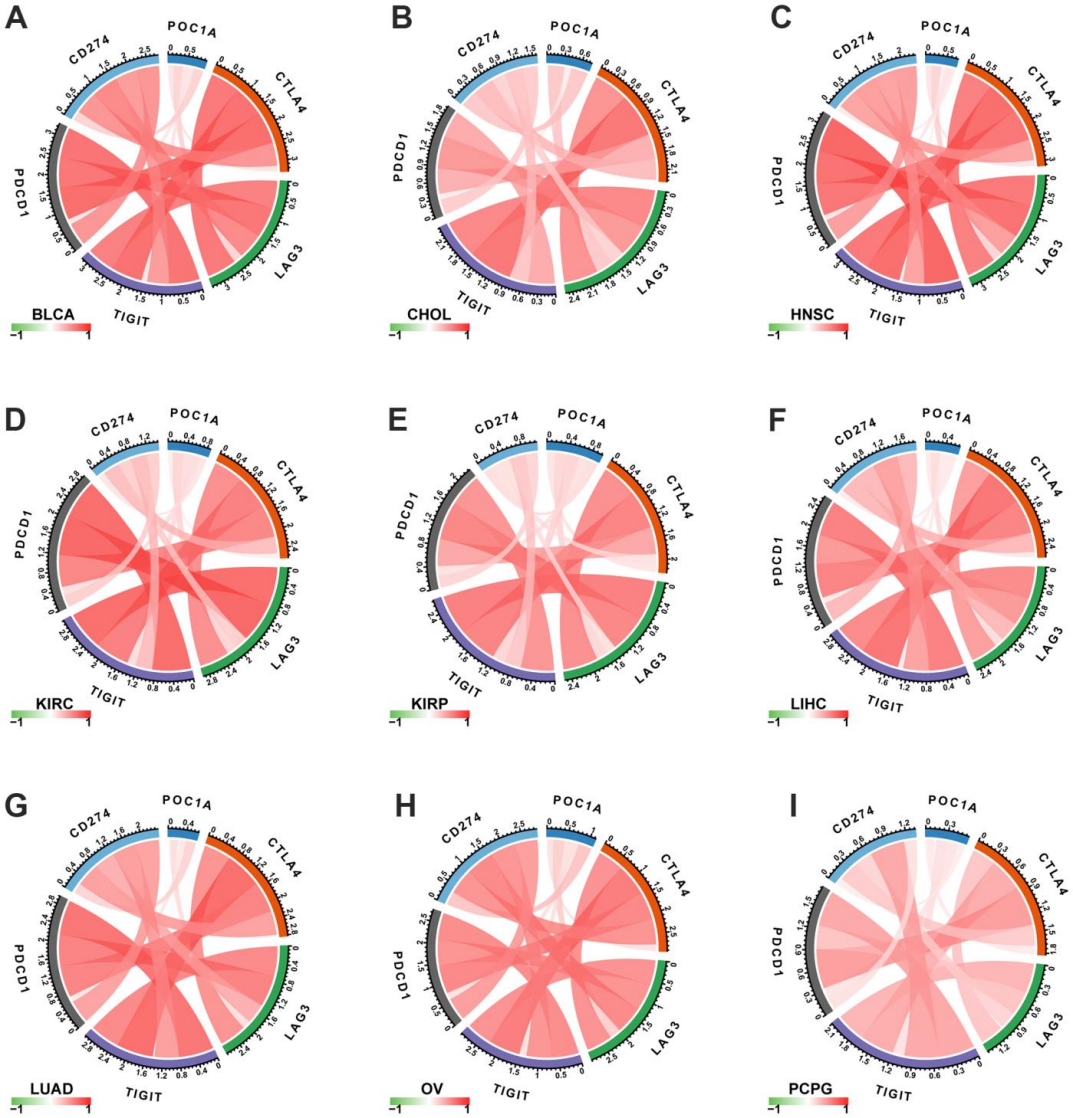
**Correlation of POC1A expression with immune checkpoint genes.** (**A**–**I**) POC1A expression is positively correlated with immune checkpoints in several tumors. Red and green lines indicate positive and negative correlations, respectively. Deeper color indicates a strong correlation.

**Figure 9:**
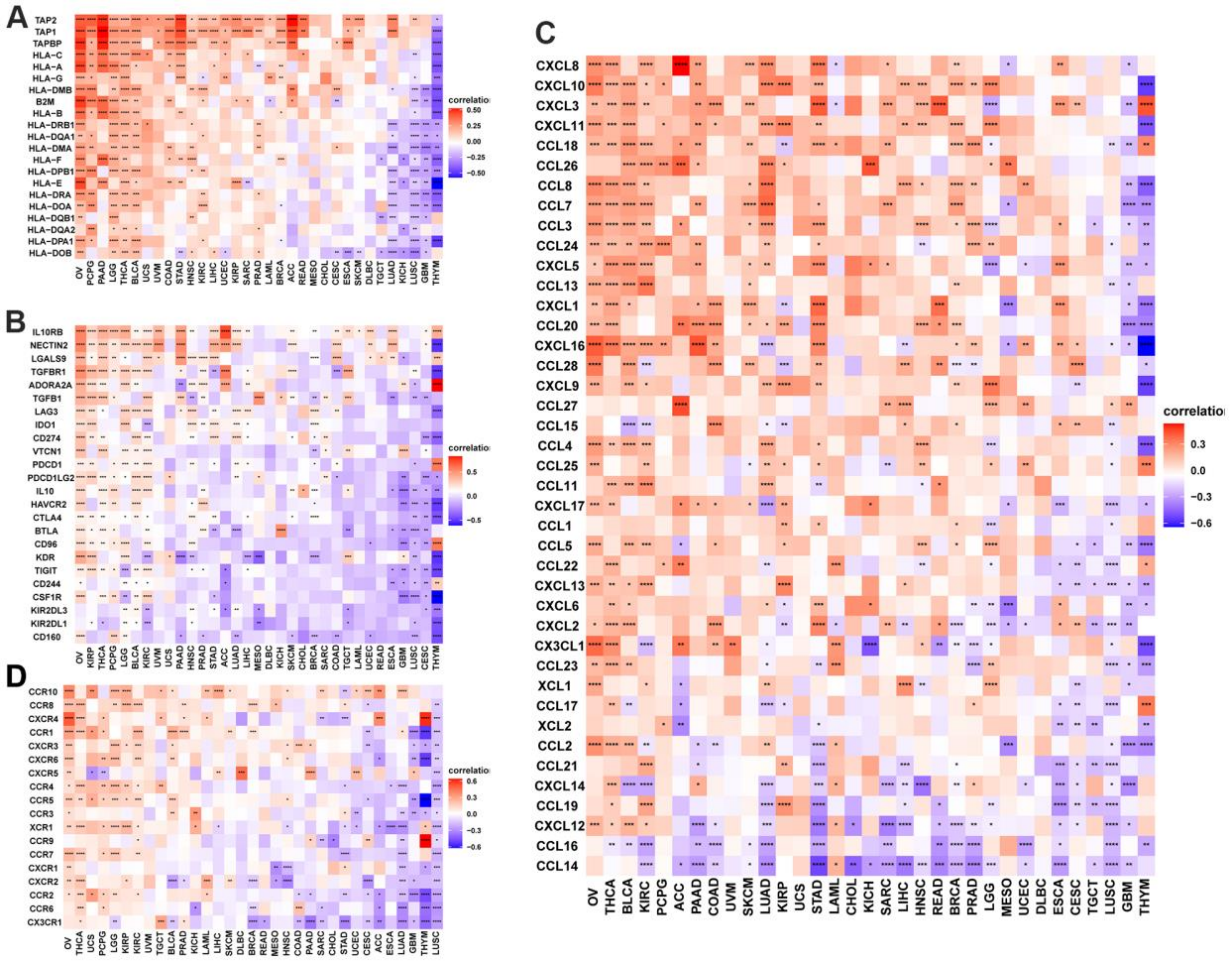
**POC1A correlation with immunomodulatory genes.** (**A**) POC1A correlation with MHC genes is represented utilizing Heatmap. (**B**) Heatmap of the POC1A correlation with immunosuppressive status-related genes. (**C**) POC1A correlation with chemokine genes is represented utilizing Heatmap. (**D**) POC1A correlation with chemokine receptor genes is represented utilizing Heatmap. R software was utilized for calculating Pearson’s correlation coefficient.

The important targets of immunotherapy are immune checkpoints genes. Five immune checkpoint genes were recognized. In pan-cancer, POC1A relation with immune checkpoint gene expression was assessed. The findings showed a positive correlation of POC1A expression with immune checkpoints in several tumors (Figure 8A–8I), suggesting that immune cells are inhibited. The correlation of POC1A with expression and immune regulatory genes was further analyzed. The results showed that the POC1A gene has a potential immunomodulatory effect in most tumors (Figure 9A–9D).

### POC1A correlation with TMB and MSI in pan-cancer

**Figure 10:**
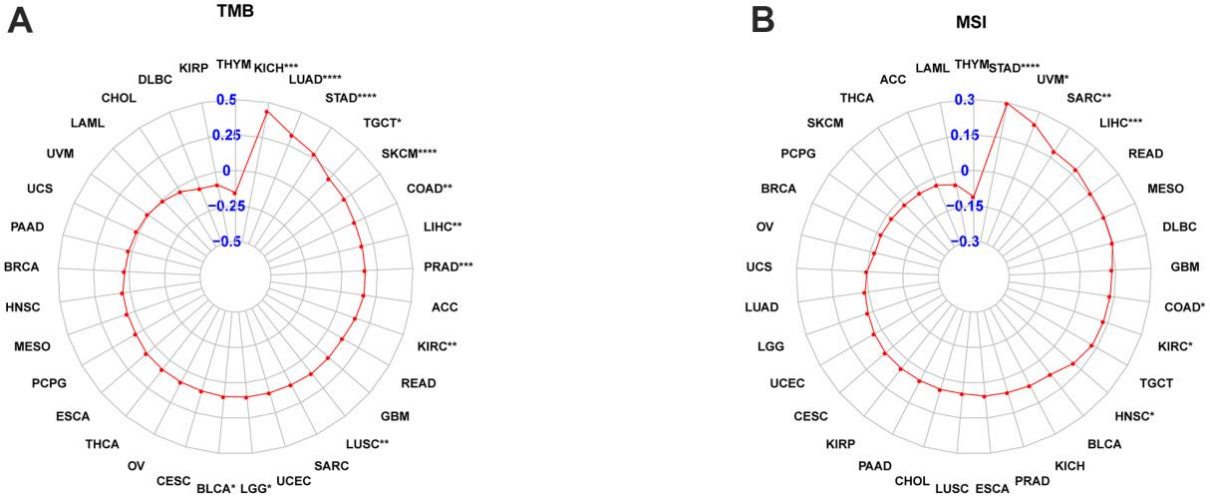
**POC1A correlation with tumor mutation burden (TMB) and microsatellite instability (MSI).** (**A**, **B**) POC1A correlation with TMB (**A**) and MSI (**B**) of Radar plots in pan-cancer. Red dots indicate the correlation coefficient. *P<0.05, **P<0.01, ***P<0.001, ****P<0.0001.

Each tumor sample’s TMB was calculated, and correlation was assessed between POC1A expression and TMB. The results are illustrated in Figure 10A. The expression levels of POC1A showed a significant positive correlation with TMB in BLCA, BRCA, COAD, GBM, KICH, LGG, LIHC, LUAD, LUSC, PAAD, PRAD, SKCM, SARC, STAD, UCEC, UCS, and UVM, and a negative correlation with TMB in THYM. The correlation of POC1A expression with MSI was assessed, and the results are illustrated in Figure 10B. Notably, POC1A expression levels had a significant positive correlation with MSI in BLCA, COAD, ESCA, HNSC, KIRC, LIHC, MESO, SARC, STAD, and UCEC, and a significant negative correlation with MSI in READ.

### GSEA analysis of POC1A

**Figure 11:**
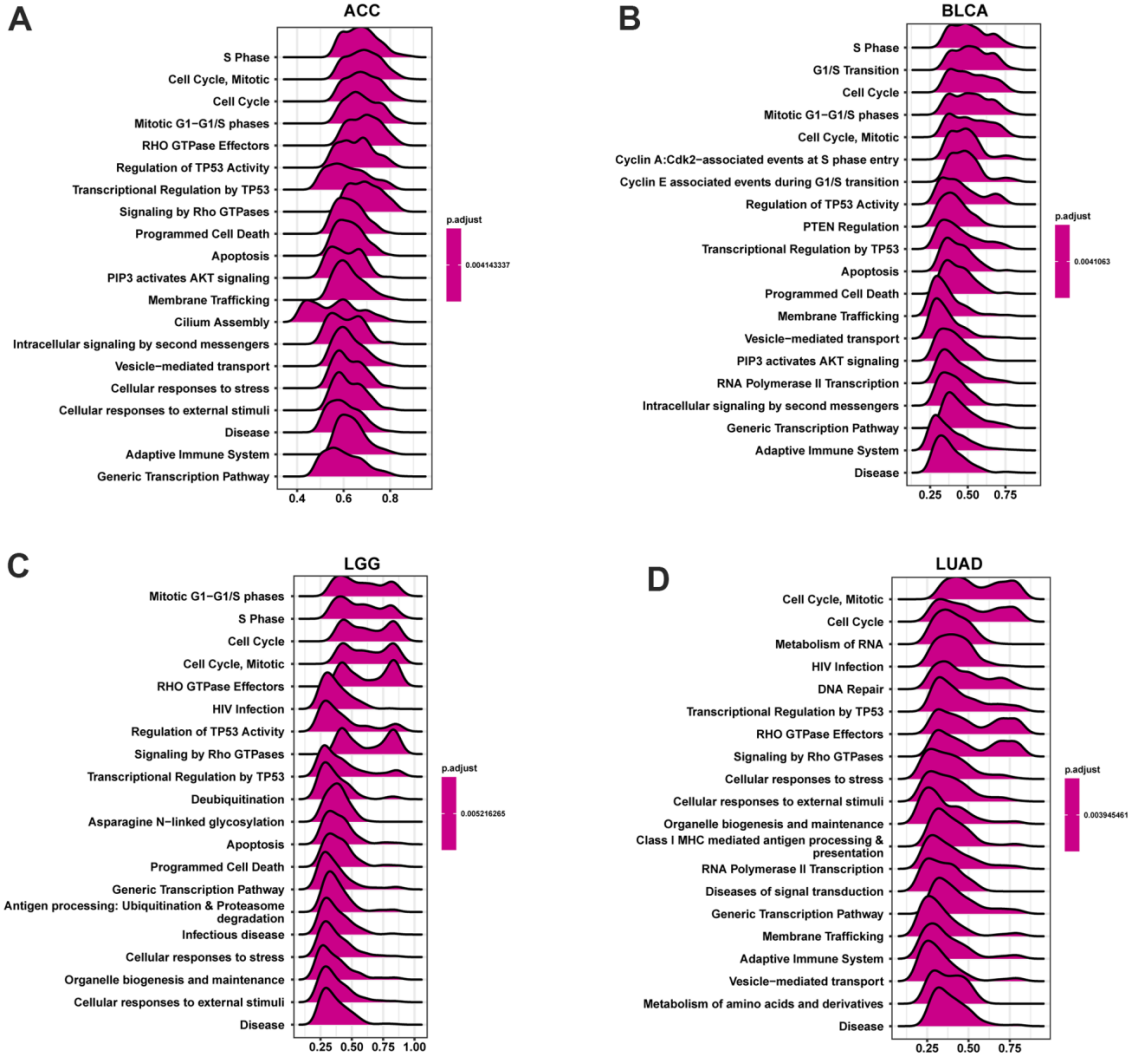
**GSEA analysis of POC1A in pan-cancer.** (**A**–**D**) GSEA detected the top twenty genes of indicated tumors (NES ≥ 1.5, adjusted P < 0.05). Red implies immune regulation-related or cell cycle-related pathways.

Based on the Reactome database, genes correlating with POC1A (P <0.05) were ranked and underwent GSEA analysis in pan-cancer. The R package “clusterProfiler” was utilized to perform the analysis. The top 20 results of each tumor identified by this analysis are shown in Figure 11. POC1A was positively correlated with immune-related and cell cycle-related pathways in various tumors, which is compatible with the previous conclusion that POC1A has an immune regulatory function.

### Drug sensitivity analysis

**Figure 12:**
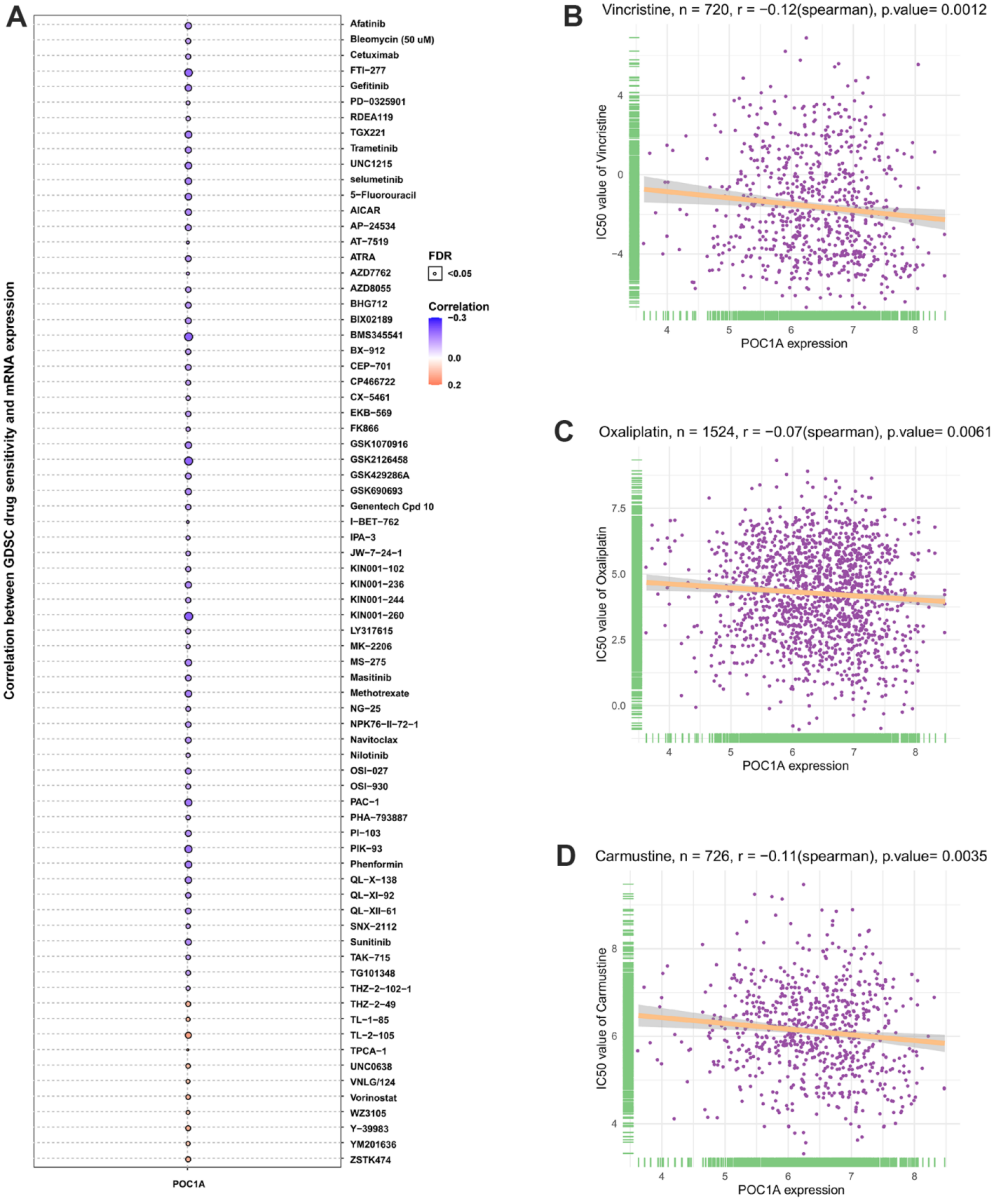
**POC1A correlation with anticancer drugs IC_50_ values.** (**A**–**D**) The correlation between POC1A expression and IC_50_ values of indicated anti-cancer drugs.

POC1A1 correlation with IC_50_ of 192 anticancer medications was evaluated. It was discovered that patients who express elevated POC1A expression might be resistant to most anticancer medications like vincristine, oxaliplatin, carmustine, etc. (Figure 12A–12D).

## DISCUSSION

The centrosome is an organelle plays a key role in cell division process and can regulate cell cycle process [18]. Several studies have affirmed that centrosome amplification is existed in practically all cancer types and has been correlated with tumorigenesis and chromosomal instability (CIN) [19–21]. Thus, abnormal centrosome regulation is a hallmark of cancer [22]. Lopes et al. studied Barrett’s esophagus patients and found that before the cells began to transform into cancer cells, they initially accumulated in the centrosomes, and centrosome expansion promoted the occurrence of esophageal cancer [23]. POC1A, an essential component of the centrosome, is known to be a cell cycle regulator. Lu et al. found that POC1A could be a potential biomarker for gastric cancer with a poor prognosis [24]. Even so, POC1A role in pan-cancer is uncertain. Therefore, the present study systematically analyzed the relation of POC1A expression with prognosis, tumor mutation burden (TMB), tumor immunity microenvironment, immune checkpoint gene, microsatellite instability (MSI) and drug sensitivity in 33 different tumors using the TCGA database.

The findings showed that POC1A was significantly highly expressed in 27 of 33 cancer types, while observed only in LAML, PCPG, and TGCT that POC1A expression was reduced. It was also found that the expression of POC1A elevated with the increase of tumor stage in nine tumor types (Figure 2A–2I). Furthermore, elevated expression of POC1A was significantly related to poor overall survival, DFI, DSS and PFI in various tumors. All of these findings imply that POC1A is an important oncogene and a potential biomarker for pan-cancer poor prognosis. Furthermore, positive correlation of POC1A mRNA expression with POC1A high CNA. Chromosome deletion of POC1A was the most marked in gastric cancer, and chromosome amplification was the most significant in seminoma. These results suggest a low level of POC1A mutation in pan-cancer and a high correlation between CNV and POC1A expression.

Current studies have illustrated that one of the causes for the poor prognosis in tumor patients is the immunosuppressive microenvironment [25–28]. Thus, we observed POC1A correlation with the immune microenvironment in pan-cancer utilizing two different immune cell infiltration data. It was noticed that POC1A expression was negatively correlated with ImmuneScore and StromalScore while positively correlated with tumor purity in majority of tumors. Besides, the infiltration levels of immune killer cells, including CD4 T and CD8 T cells and activated NK cells, were inversely correlated with POC1A expression in pan-cancer. Even so, two different analytical methods revealed a positive correlation between immunosuppressive cells, including nTregs and iTregs, and POC1A expression. POC1A correlation with the immune checkpoint gene was further evaluated. The findings revealed POC1A positive correlation with an immune checkpoint in various tumors, suggesting that immune cells were inhibited. Moreover, the correlation of POC1A expression with immunomodulatory genes was scrutinized, and the findings revealed that POC1A had potential immunomodulatory effects in most tumors (Figure 9A–9D). Collectively, these findings imply that elevated POC1A expression is related to immunosuppressive tumor microenvironment. Expression of POC1A was also significantly positively correlated with TMB and MSI in most tumors, implying that patients with elevated POC1A expression might be more susceptible to immunotherapy. Given POC1A’s role and prognostic value in pan-cancer, the possible biological function and associated signal pathway of POC1A in pan-cancer were further predicted using GSEA analysis. According to our GSEA results, POC1A was positively correlated with cell cycle and immune-related pathways in a variety of tumors. Taken together, these findings imply that the POC1A gene might have immunomodulatory functions and that tumor patients with elevated POC1A expression might be in an immunosuppressive condition.

In addition, the correlation of POC1A1 expression with the IC50 of 192 anticancer medications was carried out, and patients with elevated POC1A expression are resistant to most anticancer medications, including vincristine, oxaliplatin, carmustine, introduces a novel idea and direction for studying the mechanism of chemoresistance.

## CONCLUSIONS

This is the initial study which performed a more comprehensive POC1A bioinformatics analysis in pan-cancer. POC1A is a potential prognostic biomarker and therapeutic target in pan-cancer. Importantly, immunosuppressive tumor microenvironment and immune checkpoint genes were noticed to be related to elevated POC1A expression. We speculated that POC1A might be a novel potential biomarker during screening of immunotherapy sensitive patients.
